# Comparison of three algorithms to measure breast density on mammograms in a population-based screening cohort

**DOI:** 10.1007/s00330-026-12681-7

**Published:** 2026-06-10

**Authors:** Jim Peters, Daniëlle van der Waal, Carla H. van Gils, Mireille J. M. Broeders

**Affiliations:** 1 IQ Health Science Department, Radboud University Medical Center, Nijmegen, The Netherlands; 2 Dutch Expert Centre for Screening, Nijmegen, The Netherlands; 3 Julius Center for Health Sciences and Primary Care, University Medical Center Utrecht, Utrecht University, Utrecht, The Netherlands; 4 Department of Public Health, Erasmus MC, University Medical Center Rotterdam, Rotterdam, The Netherlands; 5 The Dutch Organization for Population Screening, Utrecht, The Netherlands; 6 ScreenPoint Medical BV, Nijmegen, The Netherlands; 7 Dutch Breast Cancer Association, Utrecht, The Netherlands

**Keywords:** Breast density, Mammography, Mass screening, Algorithms, Breast neoplasms

## Abstract

**Objectives:**

To compare breast density assessments from three automated algorithms by evaluating their agreement and associations with breast cancer risk in a large screening population.

**Materials and methods:**

We included 61,514 women from the Dutch population-based PRISMA screening cohort (2014–2019). Breast density was quantified on one screening mammogram per participant using Volpara Density Grade 4th and 5th edition, Quantra Density Assessment Software, and iCAD Density Assessment. Agreement between categorical outputs was assessed via weighted kappa statistics. Breast cancer cases were identified through linkage with the Netherlands Cancer Registry (follow-up until December 2022). Associations with five-year breast cancer risk were analyzed using Cox proportional hazards models. Discriminative ability was evaluated using the area under the receiver operating characteristic curve (AUC).

**Results:**

Good agreement was observed between algorithms (kappa: 0.725–0.826); the strongest across-vendor agreement was between Volpara Density Grade 5th edition and Quantra Density Assessment Software (kappa = 0.795). However, the proportion of women classified with extremely dense breasts (category D) varied (3.1–8.4%). During a median follow-up of 4.2 years, 777 breast cancer cases were identified. Both breast cancer risk and interval cancer risk increased with higher density, with similar magnitudes across algorithms. Categorical measures yielded similar AUCs to distinguish between women with and without breast cancer (0.53–0.55) and between screen-detected and interval cancers (0.57–0.60).

**Conclusion:**

Three automated density assessment algorithms show strong agreement and comparable associations with breast cancer risk. However, differences in categorization—particularly the proportions classified as entirely fatty or extremely dense—should be considered in personalized screening strategies, as these may influence the capacity needed for potential supplemental screening.

**Key Points:**

***Question*** *How do three automated breast density measurement algorithms compare in terms of agreement and their association with breast cancer risk in a large screening cohort*?

***Findings*** *The three algorithms showed strong agreement (kappa* = *0.725–0.826) and similar associations with breast cancer risk. Proportions of mammograms classified as density categories A or D varied*.

***Clinical relevance*** *The strong agreement and similar associations with breast cancer risk support algorithm interchangeability, but variations in the proportion of mammograms classified as entirely fatty (category A) or extremely dense (category D) may impact capacity requirements for personalized screening*.

**Graphical Abstract:**

**Figure:**
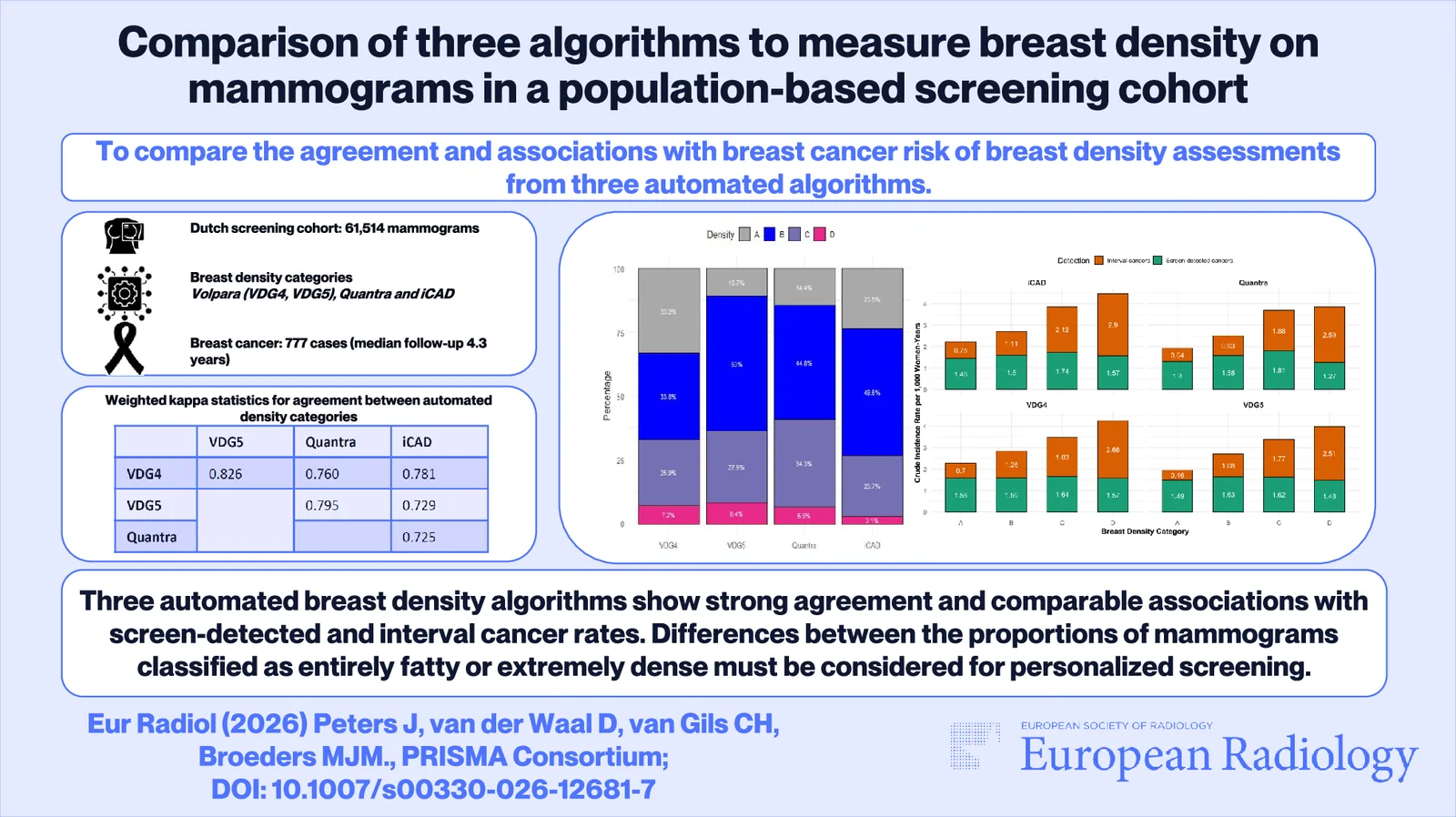

## Introduction

Breast cancer screening reduces mortality by enabling early detection and timely treatment [1]. In several countries, population-based screening programs use mammography as the primary screening modality.

Breast density—defined as the proportion of fibroglandular tissue relative to fatty tissue on a mammogram—is a key factor affecting both mammography performance and breast cancer risk. High breast density is characterized by a greater amount of fibroglandular tissue, which appears radiographically dense. Higher breast density is linked to an increased breast cancer risk [2, 3] and lowers mammographic sensitivity by masking tumors [4, 5].

This dual impact has spurred interest in incorporating breast density into personalized screening strategies [6]. Women with extremely dense breasts may benefit from shorter screening intervals or supplemental imaging like MRI to improve detection [7, 8]. Tailoring screening based on individual density profiles could enhance early detection and further reduce breast cancer mortality [9].

Breast density has traditionally been assessed visually by radiologists using BI-RADS classification. The 4th BI-RADS edition relied on percentage estimates of density, while the 5th edition uses a semi-qualitative assessment emphasizing the potential masking effect of fibroglandular tissue [10]. In both editions, however, visual evaluation is inherently subjective and prone to substantial inter-reader variability [11].

To improve consistency, automated algorithms for breast density measurement have been developed, with recent updates aligning better with BI-RADS 5th edition criteria [12, 13]. These updated tools have yet to be compared directly, and it remains unclear which provides the most accurate assessment or how their results relate to breast cancer risk. Considering masking effects, understanding differences in screen-detected and interval cancer rates across automated density categories is also important.

Using data from over 61,000 women in a Dutch prospective screening cohort, this study aims to: (1) assess the agreement of density measurements from three commercial automated algorithms (Volpara, Quantra, and iCAD), and (2) evaluate associations between these measurements and breast cancer risk, overall and separately for screen-detected and interval cancers.

## Materials and methods

### Ethics statement

The personalized risk-based mammography screening (PRISMA) study was approved by the local medical ethics committee (CMO Arnhem-Nijmegen; reference no. 2014/177). All participants provided informed consent for use of their data and mammograms; those without consent were excluded.

### Design and population

The PRISMA study is an observational, population-based, prospective cohort study, nested within the Dutch breast cancer screening program [14]. Women aged 50–75 undergo biennial mammography; those at strongly increased risk (genetic/familial history or prior thoracic irradiation) are monitored in hospital.

In the screening program, at each screening examination, a digital mammogram—including mediolateral oblique (MLO) and craniocaudal (CC) views—is acquired for each breast and read independently by two screening radiologists. Breast density is not routinely assessed.

PRISMA participants were enrolled between 2014 and 2019 in 20 screening units covering four screening regions. The distribution across regions was 55.9% North, 19.6% South West, 14.0% East, and 10.8% South. To prevent repeat participation in PRISMA, inclusion time per unit was limited to two years.

**Fig. 1:**
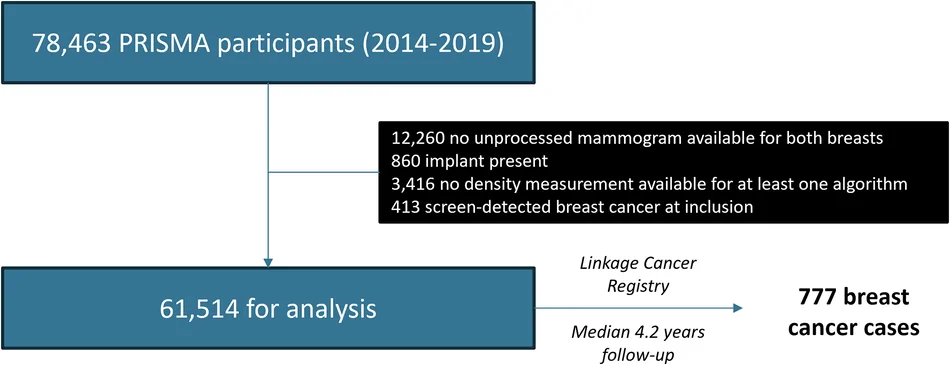
Flowchart showing the 61,514 PRISMA study participants who were included to compare the agreement of automated density measurements and their associations with breast cancer risk

Figure 1 shows the inclusion flowchart. Of 78,463 participants, we excluded those with missing unprocessed mammograms (*n* = 12,260), breast implants (*n* = 860), missing density output (*n* = 3416), and screen-detected cancer at entry (*n* = 413). Screen-detected cancers were defined as those diagnosed within 12 months of recall, and interval cancers within 30 months after a negative screen; others were non-screen-detected.

### Outcomes

The primary outcome of this study is the occurrence of breast cancer, including both in situ and invasive breast cancer. As a secondary outcome, we studied the occurrence of invasive breast cancer only. Cancer cases were identified through linkage with the Netherlands Cancer Registry, with follow-up through December 2022. The completeness of pathology-confirmed cancer diagnoses in this registry is estimated to be between 95% and 98.7% [15].

### Breast density algorithms

Breast density was assessed using the screening mammograms, both the CC and MLO views from both breasts, obtained at study entry. All mammograms were unprocessed (‘for processing’) full-field digital mammograms acquired on Hologic systems (Selenia or Selenia Dimensions). Breast density was measured using three automated algorithms: Volpara, Quantra, and iCAD.

#### Volpara

Volpara version 1.5.5.4 (Volpara Health) was used. It is a physics-based algorithm that derives tissue volumes from pixel intensities, imaging parameters, breast compression data, and expected attenuation coefficients [16]. For each mammographic view and breast, the algorithm estimates the fibroglandular volume (cm³) and total breast volume (cm³), from which volumetric breast density (VBD) % is calculated. Based on the average VBD% of both breasts, Volpara Density Grades 4th edition (VDG4) are calculated as A (< 4.5%), B (4.5%–7.5%), C (7.5%–15.5%), or D (> 15.5%). The Volpara Density Grade 5th edition (VDG5) uses the maximum VBD % of either breast and a lower threshold for category A (< 3.5% instead of < 4.5%). VDG4 and VDG5 correspond to BI-RADS 4th and 5th edition density categories, respectively.

#### Quantra

Quantra version 2.2 (Hologic Inc.) applies a machine learning–based method that leverages pattern and texture features from unprocessed digital mammograms. The model was trained on a dataset annotated by expert radiologists according to BI-RADS 5th edition criteria [12]. Quantra provides a BI-RADS–like breast density category for each breast and for the overall examination, corresponding to the BI-RADS 5th edition classification.

#### iCAD

The iCAD density assessment tool (version 2.1.0.0.64, M-Vu CAD; iCAD) estimates breast density from unprocessed mammograms by computing the total breast area (cm²) and fibroglandular tissue area (cm²). The ratio of these areas yields the breast density percentage for each breast. The software also assigns BI-RADS–like density categories (A–D) consistent with the BI-RADS 5th edition.

### Statistical methods

#### Agreement between methods

Because not all algorithms yield continuous breast density measures, agreement was primarily assessed using categorical outputs. For each pair of the three algorithms, agreement was quantified by absolute agreement (i.e., the proportion of mammograms categorized identically) and weighted kappa statistics with 95% confidence intervals (CIs), using quadratic weighting. Kappa values were interpreted using Altman’s modification of the Landis and Koch scale: poor (≤ 0.20), fair (0.21–0.40), moderate (0.41–0.60), good (0.61–0.80), and very good (> 0.80) [17, 18]. The weighted kappa accounts for the magnitude of disagreement when categories assigned by two algorithms differ by more than one level. Bangdiwala’s agreement charts were used for visual assessment of concordance between methods [19]. Volpara and iCAD also produce continuous density measures (VBD%, maximum VBD%, and percent dense tissue), but these are defined differently and not directly comparable. Therefore, instead of agreement statistics, we evaluated correlations using Spearman’s coefficient.

#### Associations with breast cancer risk and screen-detected and interval cancers

Associations between breast density and five-year breast cancer risk were evaluated using both categorical and continuous density measures. Kaplan–Meier curves were constructed to visualize cumulative breast cancer incidence across density categories for each algorithm. To understand how mammographic sensitivity would differ across density categories defined by the different algorithms, crude incidence rates of screen-detected and interval cancers were calculated separately across density categories.

Univariable Cox proportional hazards models were used to estimate hazard ratios (HRs) with 95% CIs. The proportional hazards assumption was verified via visual inspection of scaled Schoenfeld residuals and found not to be violated. Continuous density measures (VBD%, Maximum VBD% and percent dense tissue) were log-transformed and standardized before inclusion in the model. For categorical density measurements, category B served as the reference.

Density categories A and D are of particular interest for personalized screening, as they may trigger adapted screening recommendations. To assess whether disagreement between algorithms influences risk estimates, HRs for categories A and D (vs B) for each algorithm were re-estimated, restricting category A or D to women whose density was classified differently by at least one other algorithm (that is, higher than A or lower than D, respectively, regardless of the number of categories that they were discordant. All B-category women were retained as the reference group in both analyses.

To compare the algorithms with each other, the discriminatory performance of each density metric, both the categorical and continuous ones, was also summarized by calculating the 5-year area under the receiver operating characteristic curve (AUC) for breast cancer. Finally, to understand the ability of the different density metrics to discriminate between cancers that are screen-detected and those that are not, e.g., due to masking, we calculated a 5-year AUC for screen-detected vs interval cancers.

All analyses in this study are complete case analyses and performed in R version 4.4.3.

## Results

### Baseline characteristics and density distribution

A total of 61,514 mammographic exams from an equal number of PRISMA participants were included in the analysis (Fig. 1). The median age at the time of screening was 60.1 years (interquartile range (IQR): 54.5–66.6).

**Fig. 2:**
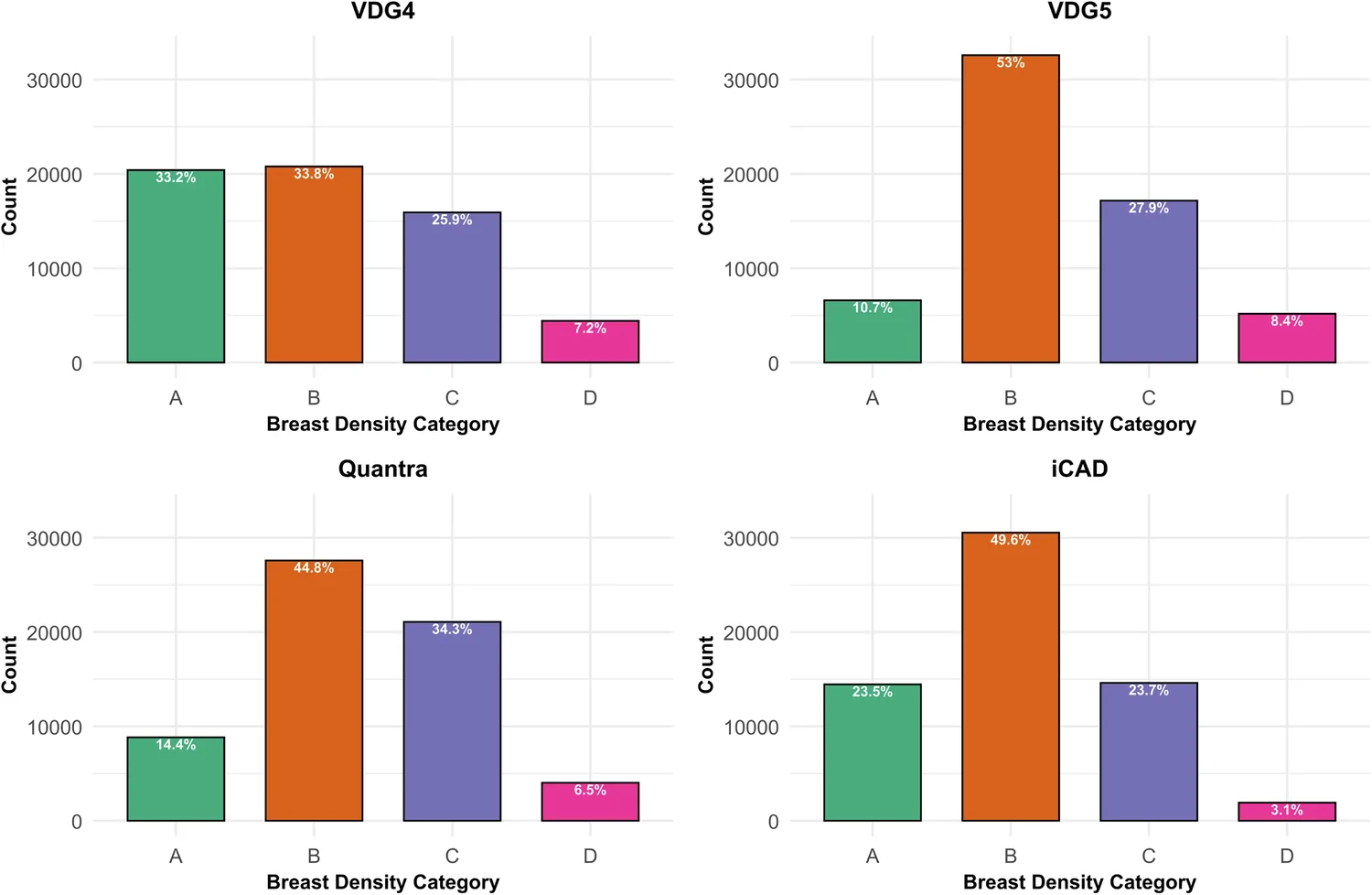
Distributions of automatically measured breast density categories using three algorithms in the PRISMA cohort (*N* = 61,514)

Figure 2 presents the distribution of breast density categories according to the four classification methods: VDG4, VDG5, Quantra, and iCAD. For the highest density category (category D), the number and percentage of exams classified as such were 5177 (8.4%) with VDG5, 4418 (7.2%) with VDG4, 4024 (6.5%) with Quantra, and 1916 (3.1%) with iCAD. The number and percentage of exams classified in the lowest density category (category A) were 20,400 (33.2%) with VDG4, 14,451 (23.5%) with iCAD, 8834 (14.4%) with Quantra, and 6594 (10.7%) with VDG5. The median continuous % breast density measures were 5.6% (IQR: 4.0%–8.8%) for VBD% (Volpara), 6.0% (IQR: 4.2%–9.4%) for Maximum VBD% (Volpara), and 9.5% (IQR: 3.0%–26.0%) for percent dense tissue (iCAD).

### Agreement between algorithms

**Fig. 3:**
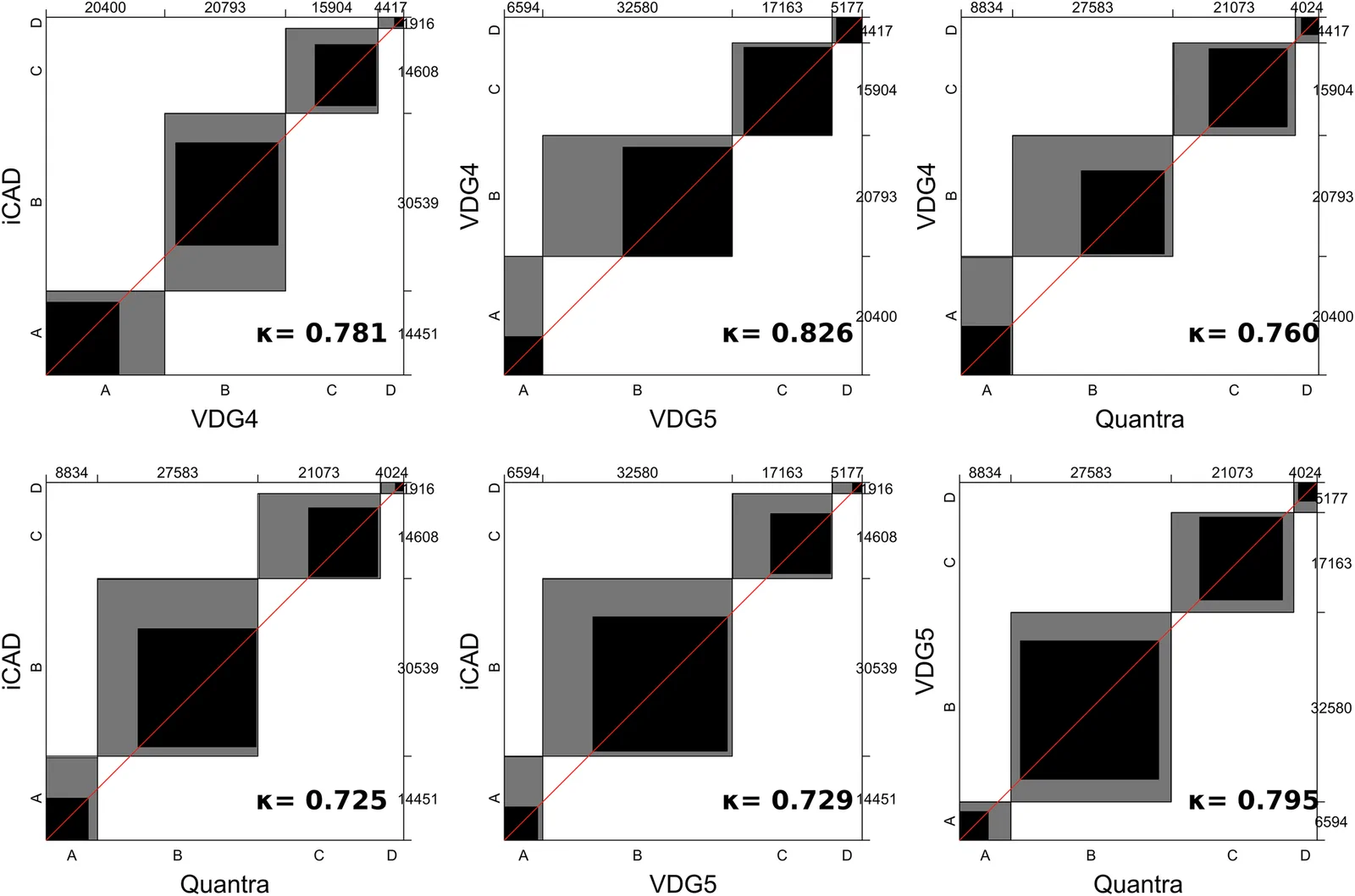
Agreement charts to compare breast density categorization on mammograms for three different algorithms in the PRISMA cohort. Black areas indicate agreement, gray areas and their orientation indicate disagreement and its direction. Weighted Fleiss kappa represents overall agreement

Tables 1 and 2 quantify the agreement between the categorical breast density measures, with visual representation provided in Fig. 3. Although disagreements between methods were observed in all directions, disagreements spanning more than one category were infrequent. Weighted kappa values ranged from 0.725 to 0.826, indicating good agreement across all comparisons.

**Table 1:** Cross-tabulation to compare breast density categorization on mammograms for three different algorithms in the PRISMA cohort

		VDG5				Quantra				iCAD			
		A	B	C	D	A	B	C	D	A	B	C	D
VDG4	A	6594 (10.6%)	13,806 (22.3%)	0 (0.0%)	0 (0.0%)	8380 (13.6%)	11,826 (19.1%)	185 (0.3%)	9 (0.0%)	12,507 (20.2%)	7881 (12.8%)	12 (0.0%)	0 (0.0%)
	B	0 (0.0%)	18,761 (30.4%)	2019 (3.3%)	0 (0.0%)	438 (0.7%)	14,265 (23.1%)	6031 (9.8%)	46 (0.1%)	1888 (3.1%)	17,581 (28.5%)	1299 (2.1%)	12 (0.0%)
	C	0 (0.0%)	13 (0.0%)	15,143 (24.5%)	760 (1.2%)	10 (0.0%)	1475 (2.4%)	13,444 (21.8%)	987 (1.6%)	56 (0.1%)	5033 (8.1%)	10,496 (17.0%)	331 (0.5%)
	D	0 (0.0%)	0 (0.0%)	1 (0.0%)	4417 (7.1%)	6 (0.0%)	17 (0.0%)	1413 (2.3%)	2982 (4.8%)	0 (0.0%)	44 (0.1%)	2801 (4.5%)	1573 (2.5%)
VDG5	A					4920 (8.0%)	1632 (2.6%)	40 (0.1%)	2 (0.0%)	5723 (9.3%)	871 (1.4%)	0 (0.0%)	0 (0.0%)
	B					3893 (6.3%)	23,785 (38.5%)	4859 (7.9%)	43 (0.1%)	8639 (14.0%)	23,077 (37.4%)	856 (1.4%)	8 (0.0%)
	C					14 (0.0%)	2137 (3.5%)	14,233 (23.0%)	779 (1.3%)	89 (0.1%)	6517 (10.6%)	10,302 (16.7%)	255 (0.4%)
	D					7 (0.0%)	29 (0.0%)	1941 (3.1%)	3200 (5.2%)	0 (0.0%)	74 (0.1%)	3450 (5.6%)	1653 (2.7%)
Quantra	A									7219 (11.7%)	1607 (2.6%)	1 (0.0%)	7 (0.0%)
	B									6982 (1.1%)	20,296 (32.8%)	298 (0.5%)	7 (0.0%)
	C									240 (0.4%)	8502 (13.8%)	11,859 (19.2%)	472 (0.8%)
	D									10 (0.0%)	134 (0.2%)	2450 (4.0%)	1430 (2.3%)

Absolute numbers of mammograms and percentages of the total number of mammograms are given

**Table 2:** Absolute agreement and weighted kappa statistics for agreement between automated breast density categories measured with three different algorithms in the PRISMA cohort

	VDG5		Quantra		iCAD	
	Absolute agreement (%)	Weighted kappa [95% CI]	Absolute agreement (%)	Weighted kappa [95% CI]	Absolute agreement (%)	Weighted kappa [95% CI]
VDG4	73.0	0.826 [0.824–0.829]	63.5	0.760 [0.756–0.763]	68.5	0.781 [0.778–0.784]
VDG5			75.0	0.795 [0.791–0.797]	66.3	0.729 [0.726–0.732]
Quantra					66.3	0.725 [0.721–0.729]

Comparing measures from different vendors, the strongest agreement was observed between VDG5 and Quantra, both calibrated to BI-RADS 5th edition. The absolute agreement between these algorithms was 75.0%, with a weighted kappa of 0.795 (95% CI: 0.791–0.797). In cases classified as category D by VDG5, a substantial proportion (*n* = 1941) were downgraded to category C by Quantra, while fewer (*n* = 779) were upgraded from C to D. At the lower end of the spectrum, more exams were classified as category B by VDG5 and as category A by Quantra (*n* = 3893) than the reverse (*n* = 1632).

iCAD showed the strongest agreement with VDG4, with an absolute agreement of 66.3% and a weighted kappa of 0.781 (95% CI: 0.778–0.784). Among the 4705 exams classified as category D by either algorithm, most discrepancies involved downgrading by iCAD to category C (*n* = 2801), with only 331 exams upgraded from C to D by iCAD. Similarly, more exams were classified as category B by iCAD while VDG4 classified them as category A (*n* = 7881), compared to the reverse situation (*n* = 1888).

Spearman correlations between continuous breast density measures were strong across vendors: 0.903 [95% CI: 0.901-0.904] between VBD % and percent dense tissue, and 0.898 [95% CI: 0.896-0.900] between Maximum VBD % and percent dense tissue.

### Associations with breast cancer risk, screen-detected, and interval cancers

**Fig. 4:**
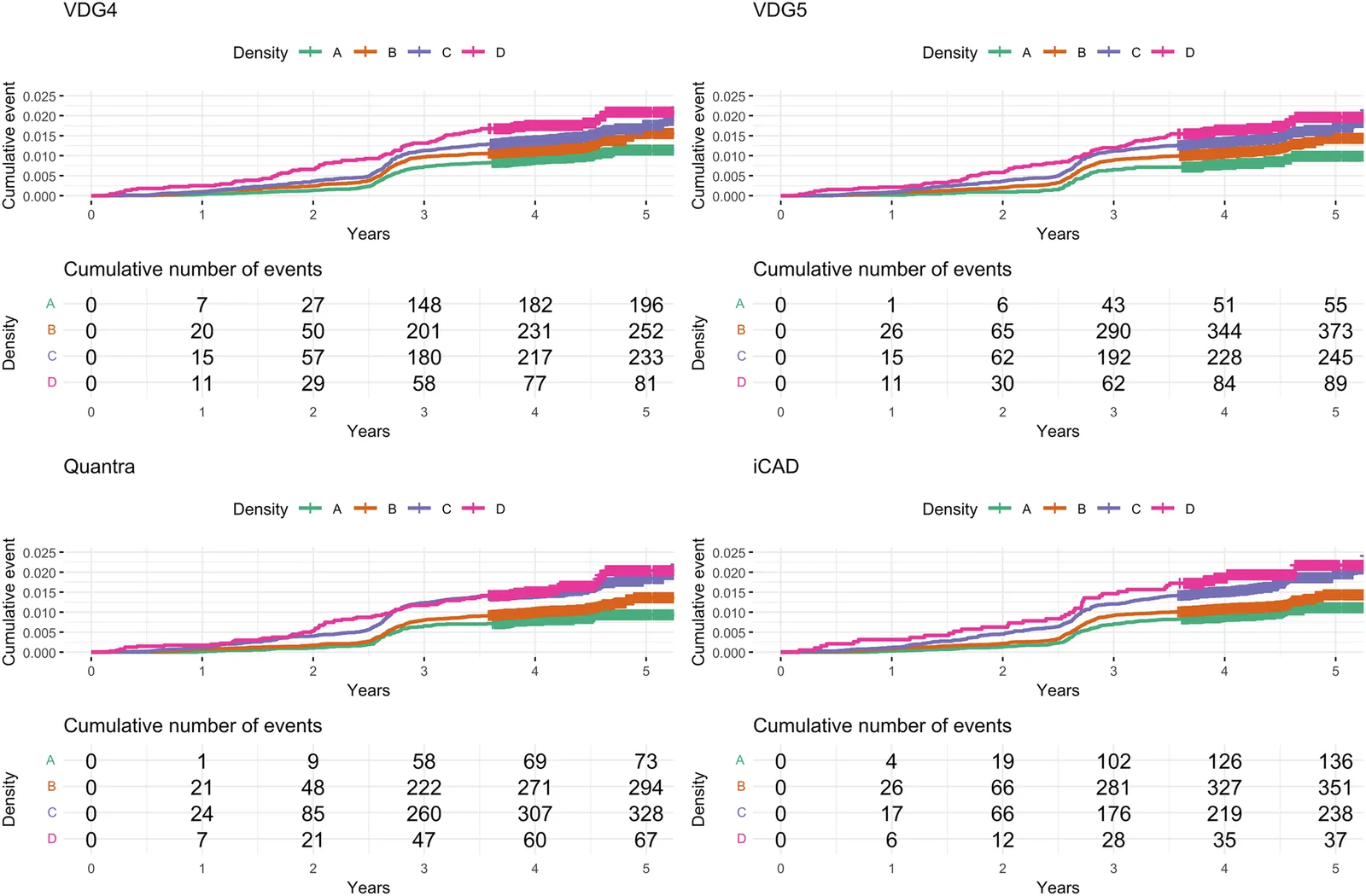
Stratified cumulative breast cancer incidence (proportion) curve by breast density category for three different automated breast density algorithms. Risk tables display the cumulative number of events across breast density levels

During a median follow-up of 4.2 years (IQR: 3.9–4.3), a total of 777 breast cancer cases were identified, of which 762 occurred within the first five years. Of those, 419 were screen-detected cancers, 317 were interval cancer and 26 were non-screen-detected. Figure 4 displays cumulative breast cancer incidence curves stratified by density category for each of the categorical breast density algorithms. The cumulative absolute number of breast cancer cases per density category for each algorithm is displayed in the risk tables in Fig. 4. It is important to note that the number of women in each density category varies depending on the classification method (Fig. 2). Consequently, the absolute number of breast cancer cases in the highest density category (category D) differed across algorithms, ranging from 37 cases among 1916 participants classified as D by iCAD, to 89 cases among 5177 participants classified as D by VDG5. Similarly, in the lowest density category A, the absolute number of cases ranged from 55 among 6594 participants classified as A by VDG5 to 196 among 20,793 participants classified as A by VDG4.

Despite these differences in category size, the cumulative incidence curves revealed similar event rates across all methods (Fig. 4). Table 3 shows that breast cancer risk consistently increased with higher breast density across all categorical measures. The three continuous measures also demonstrated similar associations with breast cancer risk: the HR per one-standard-deviation increase in log-transformed density % was 1.22 (95% CI: 1.14–1.30) for VBD %, 1.22 (95% CI: 1.14–1.31) for maximum VBD %, and 1.29 (95% CI: 1.19–1.38) for percent dense tissue.

**Table 3:** Associations of breast density measures with breast cancer risk and five-year area under the receiver operating curves to discriminate between participants with and without breast cancer, and to discriminate between screen-detected and interval cancers in the PRISMA cohort

		Associations with breast cancer risk		Discriminatory accuracy			
				Breast cancer vs no breast cancer		Screen-detected vs interval cancer	
Breast density measure		HR	95% CI	Area under the curve	95% CI	Area under the curve	95% CI
VDG4	A	0.80	0.66–0.96	0.53	0.52–0.55	0.57	0.53–0.61
	B	Ref	Ref				
	C	1.22	1.02–1.46				
	D	1.49	1.16–1.91				
VDG5	A	0.72	0.54–0.96	0.54	0.52–0.56	0.58	0.54–0.62
	B	Ref	Ref				
	C	1.25	1.07–1.47				
	D	1.47	1.17–1.85				
Quantra	A	0.77	0.60–0.99	0.55	0.53–0.57	0.60	0.56–0.63
	B	Ref	Ref				
	C	1.46	1.26–1.72				
	D	1.53	1.18–2.00				
iCAD	A	0.82	0.67–1.00	0.54	0.52–0.56	0.57	0.54–0.61
	B	Ref	Ref				
	C	1.43	1.21–1.68				
	D	1.65	1.18–2.32				
VBD % (Volpara)	%	1.22	1.14–1.30	0.57	0.55–0.59	0.62	0.58–0.66
Max. VBD % (Volpara)	%	1.22	1.14–1.31	0.56	0.54–0.58	0.62	0.58–0.66
Percent dense tissue % (iCAD)	%	1.29	1.19–1.38	0.56	0.54–0.58	0.60	0.56–0.64

Continuous measures were log-transformed and normalized

The HRs presented in Table 4 show that, for participants with breast density category A or D according to a given algorithm, associations with breast cancer risk were not different in case of disagreement with one or more of the other algorithms (i.e., classified as discordant).

**Table 4:** Associations of categorical breast density measures A and D with breast cancer, for all participants categorized as A or D by an algorithm, or considering only discordant measurements

			Associations with breast cancer risk	
Breast density measure			HR	95% CI
VDG4	A	All (*N* = 20,400)	0.80	0.66–0.96
		Discordant (*N* = 15,917)	0.84	0.69–1.02
	B	All (*N* = 20,793)	Ref	Ref
	D	All (*N* = 4417)	1.49	1.16–1.91
		Discordant (*N* = 3101)	1.50	1.16–1.92
VDG5	A	All (*N* = 6594)	0.72	0.54–0.96
		Discordant (*N* = 2171)	0.76	0.48–1.20
	B	All (*N* = 32,580)	Ref	Ref
	D	All (*N* = 5177)	1.47	1.17–1.85
		Discordant (*N* = 3861)	1.46	1.13–1.90
Quantra	A	All (*N* = 8834)	0.77	0.60–0.99
		Discordant (*N* = 4411)	0.78	0.55–1.10
	B	All (*N* = 27,583)	Ref	Ref
	D	All (*N* = 4024)	1.53	1.18–2.00
		Discordant (*N* = 2708)	1.50	1.09–2.06
iCAD	A	All (*N* = 14,451)	0.82	0.67–1.00
		Discordant (*N* = 10,028)	0.87	0.70–1.09
	B	All (*N* = 30,539)	Ref	Ref
	D	All (*N* = 1916)	1.65	1.18–2.32
		Discordant (*N* = 600)	2.02	1.18–3.44

**Fig. 5:**
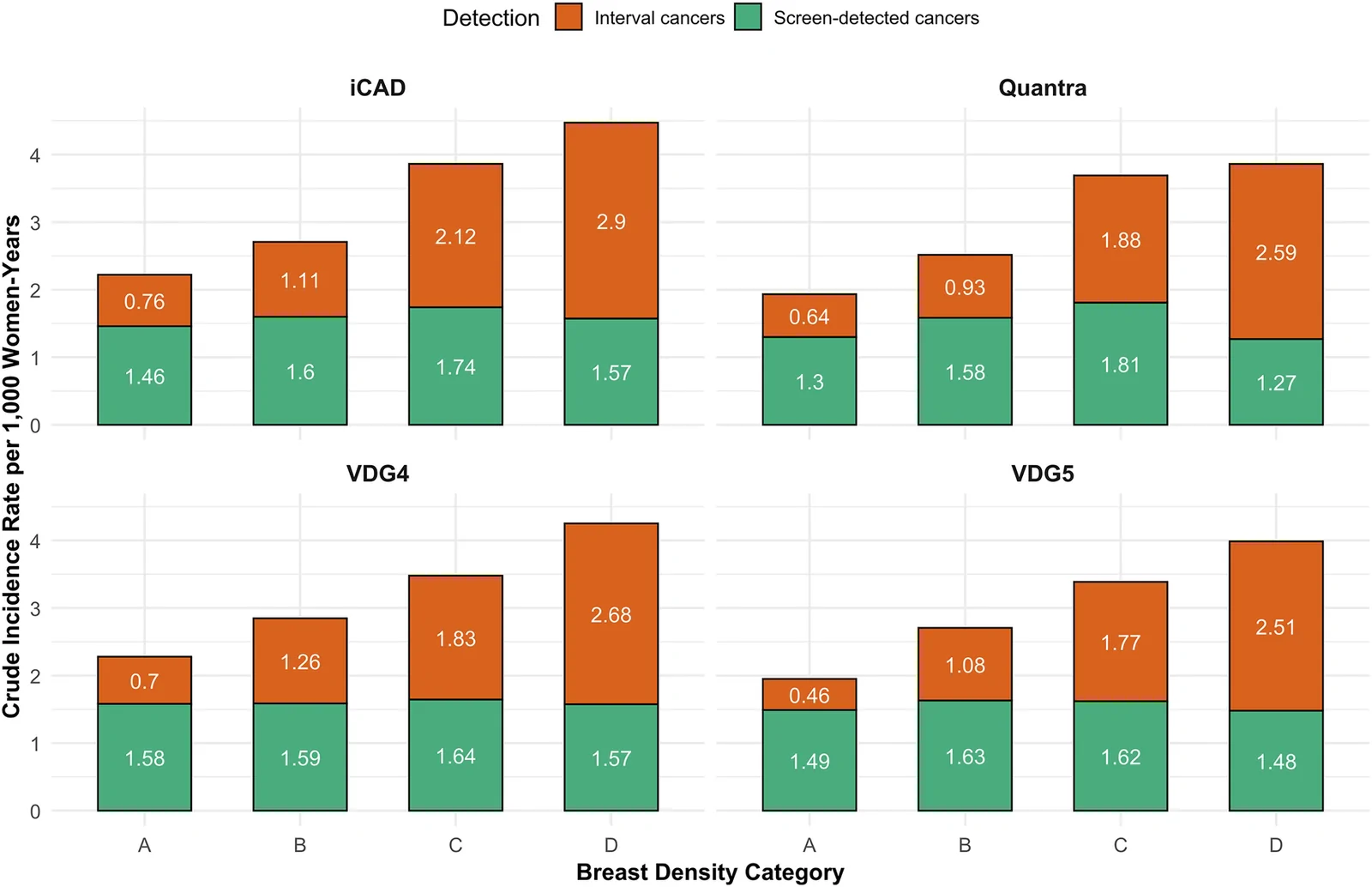
Crude screen-detected and interval cancer rates per 1000 women-years by density categories for three different breast density algorithms in the PRISMA cohort

Figure 5 shows crude incidence rates per 1000 women-years for screen-detected and interval cancers separately. For all algorithms, the interval cancer rates increase relative to the screen-detected cancer rates as density increases. These increases are of similar magnitude across the three algorithms.

Regarding discriminatory performance, the five-year area under the curve (AUC) estimates to distinguish between women with and without breast cancer were similar among categorical measures: 0.53 (95% CI: 0.52–0.55) for VDG4, 0.54 (95% CI: 0.52–0.55) for VDG5, 0.55 (95% CI: 0.53–0.57) for Quantra, and 0.54 (95% CI: 0.52–0.56) for iCAD. The continuous density measures showed slightly higher discrimination: 0.57 (95% CI: 0.55–0.59) for VBD %, 0.56 (95% CI: 0.54–0.58) for Maximum VBD %, and 0.56 (95% CI: 0.54–0.58) for percent dense tissue. Table 3 also shows AUC estimates to distinguish between women with screen-detected and interval cancer. For categorical metrics, the AUCs range from 0.57 (VDG4) to 0.60 (Quantra). Again, slightly higher AUCs are observed (up to 0.62) for the continuous density measures.

Of the 762 breast cancer cases in the first 5 years of follow-up, 657 were invasive. Table S1 shows that associations with invasive breast cancer risk for all density measures are similar to the associations with overall breast cancer risk (Table 3).

## Discussion

### Summary of findings

In this large, population-based cohort of over 61,000 women participating in the Dutch breast cancer screening program, we evaluated breast density using three automated algorithms. We compared their agreement, and association with breast cancer risk, as well as screen-detected and interval cancer rates. The algorithms demonstrated good agreement, with weighted kappa values ranging from 0.725 to 0.826 between categorical measures. Across vendors, the strongest agreement was observed between VDG5 and Quantra.

Breast cancer incidence rose consistently with increasing density across all algorithms, and all three continuous measures were significantly associated with five-year risk. Discrimination was modest for every measure, with only a small AUC improvement when using continuous instead of categorical density (from ~0.53–0.55 to ~0.56–0.57). Screen-detected vs interval cancer patterns were likewise similar across density algorithms.

Although overall agreement between algorithms was strong, they assigned different proportions of women to categories A and D. In personalized screening, this would influence both the size of risk strata and the number of cancers within them. Because cumulative risks were comparable, variation in absolute case counts mainly reflects differences in category distribution rather than underlying risk.

### Literature comparison

To the best of our knowledge, this is the largest study to date comparing agreement between multiple automated breast density algorithms on screening mammograms. While several studies have evaluated breast density algorithms, direct comparison with our findings is limited, primarily because only the version of Volpara used in this study has been evaluated in previous research. Comparative studies including iCAD’s density measurement are currently lacking [20].

Prior research has largely focused on comparisons between Volpara and earlier versions of Quantra, which provided continuous volumetric density outputs [13, 21–25]. A recent meta-analysis reported strong agreement between the volumetric breast density estimates of these two algorithms, with a pooled correlation coefficient of 0.839 [20]. Other automated or semi-automated density measurement tools, such as Cumulus and LIBRA, have also shown strong agreement with each other and similar associations with breast cancer risk in a previous study [26].

Although Quantra version 2.2 adopts a different approach—using a multi-class classification model instead of volumetric estimation—our results show similarly strong agreement between its categorical output and that of Volpara and thus align with previous findings that used other versions.

Regarding breast cancer risk, the increasing HRs across breast density categories observed here align with earlier findings, reinforcing breast density as an important risk factor. Its impact on mammographic sensitivity, likely via masking, is confirmed by our results, which agree with studies showing increasing interval cancer rates as density increases [4, 5, 27, 28].

### Clinical implications

The strong agreement between the three automated breast density algorithms, along with similar associations with breast cancer risk and comparable screen-detected and interval cancer rates, suggests no single algorithm clearly outperforms the others in this setting. This is reassuring, implying different tools may be used interchangeably to support breast density assessment, for example, in risk prediction for tailored screening. Moreover, our findings suggest that treating breast density as a continuous rather than a categorical measure may provide incremental value. The slightly higher discriminatory performance of continuous measures, reflected in AUCs, supports their potential utility in risk models.

Despite strong agreement and similar risk associations, notable differences in the proportion of women classified into the lowest or highest density categories (“A” and “D”) must be carefully considered, especially for personalized screening. Since category D may require the decision to intensify screening (e.g., supplemental imaging, shorter screening interval), variability in classification could lead to substantial differences in the number of women selected for additional interventions, depending on the algorithm, which influences the required capacity. Similarly, if de-intensified screening (e.g., extended intervals) is considered for category A, the choice of algorithm affects the number of women eligible. This also influences the absolute number of cases in these groups, affecting the potential benefit of different screening strategies.

### Limitations

Several limitations should be acknowledged. First, although various commercial breast density algorithms exist, only three were included. Still, these represent distinct technical approaches covering most methodologies used in other tools [20]. One approach not represented is deep learning–based density classification. For example, Eun Lee et al compared an AI-based and physics-based model to radiologists’ assessments in 488 Asian women, finding similar agreement of both models and radiologists [29].

Second, all mammograms were acquired using a single vendor (Hologic), limiting generalizability across mammography systems. We could not assess if agreement varies by system. However, prior work found system choice did not significantly impact density estimates [30].

Another important limitation, not unique here, is the absence of a gold standard for breast density measurement, preventing definitive conclusions on any algorithm’s validity. Some studies compare volumetric mammographic density to MRI, especially for volumetric algorithms, while others focus on predicting BI-RADS categories [31]. The assessment of an algorithm’s validity may depend on the chosen target metric. Agreement between automated algorithms and radiologists’ BI-RADS is generally moderate, likely reflecting inter-reader variability. Interestingly, agreement between automated algorithms tends to be higher due to their inherent consistency [20].

In the absence of a gold standard, we assessed associations between each density metric and breast cancer incidence, and compared screen-detected and interval cancer rates. Although longer follow-up would strengthen conclusions, these outcomes are clinically meaningful, especially considering the use of breast density to guide personalized screening strategies.

Finally, the PRISMA cohort may not be fully representative of the entire Dutch screening population. However, it covers multiple regions with good geographic representation, and the age distribution closely reflects the target screening population [32].

## Conclusion

In this large population-based screening cohort, three automated breast density algorithms showed good agreement and comparable associations with breast cancer risk, as well as with screen-detected and interval cancer rates, suggesting they may be used interchangeably to support density assessment. Differences in the proportion of women classified as entirely fatty or extremely dense, however, should be considered when implementing personalized screening as they directly affect the number of women eligible for de-intensified or supplemental screening and the capacity required to deliver it.

## Supplementary information

Table S1: Associations of breast density measures with invasive breast cancer risk and five-year area under the receiver operating curves to discriminate between participants with and without invasive breast cancer. Continuous measures were log-transformed and normalized.

## Data Availability

The data underlying this study are not publicly available due to participant privacy and consent restrictions. Participants provided consent for data sharing with other researchers under defined conditions, including re-use of data for research on breast cancer. Parts of the data are provided by third parties, the Netherlands Comprehensive Cancer Organization and Stichting Bevolkingsonderzoek Nederland, under license. Researchers interested in accessing the data can contact the corresponding author to submit a request, which will be reviewed by the PRISMA Data Access Committee. For researchers who meet the criteria, data can be shared with permission of the third parties.

## Abbreviations

AUC: Area under the receiver operating characteristic curve
CC: Craniocaudal
CI: Confidence interval
HR: Hazard ratio
IQR: Interquartile range
MLO: Mediolateral oblique
PRISMA: Personalized risk-based mammography screening
VDG5: Volpara density grade 5th edition

## References

[1] Zielonke N, Gini A, Jansen EE et al (2020) Evidence for reducing cancer-specific mortality due to screening for breast cancer in Europe: a systematic review. Eur J Cancer 127:191–206 10.1016/j.ejca.2019.12.010 31932175

[2] McCormack VA, dos Santos Silva I (2006) Breast density and parenchymal patterns as markers of breast cancer risk: a meta-analysis. Cancer Epidemiol Biomark Prev 15:1159–1169 10.1158/1055-9965.EPI-06-0034 16775176

[3] Pettersson A, Graff RE, Ursin G et al (2014) Mammographic density phenotypes and risk of breast cancer: a meta-analysis. J Natl Cancer Inst 106:dju078 10.1093/jnci/dju078 PMC4568991 24816206

[4] Wanders JO, Holland K, Veldhuis WB et al (2017) Volumetric breast density affects performance of digital screening mammography. Breast Cancer Res Treat 162:95–103 10.1007/s10549-016-4090-7 PMC5288416 28012087

[5] Payne NR, Hickman SE, Black R, Priest AN, Hudson S, Gilbert FJ (2025) Breast density effect on the sensitivity of digital screening mammography in a UK cohort. Eur Radiol 35:177–187 10.1007/s00330-024-10951-w PMC11631811 39017933

[6] Caumo F, Gennaro G, Ravaioli A et al (2025) Personalized screening based on risk and density: prevalence data from the RIBBS study. La Radiologia Medica 130:740–752 10.1007/s11547-025-01981-5 PMC12106505 40117106

[7] Bakker MF, de Lange SV, Pijnappel RM et al (2019) Supplemental MRI screening for women with extremely dense breast tissue. N Engl J Med 381:2091–2102 10.1056/NEJMoa1903986 31774954

[8] Mann RM, Athanasiou A, Baltzer PA et al (2022) Breast cancer screening in women with extremely dense breasts recommendations of the European Society of Breast Imaging (EUSOBI). Eur Radiol 32:4036–4045 10.1007/s00330-022-08617-6 PMC9122856 35258677

[9] Gil Quessep E, van der Waal D, Peters J et al (2025) Subgroups varying in risk and density highlight the potential for stratified breast cancer screening. Eur J Cancer 217:115220 10.1016/j.ejca.2025.115220 PMC13067045 39805236

[10] Spak DA, Plaxco J, Santiago L, Dryden M, Dogan B (2017) BI-RADS® fifth edition: a summary of changes. Diagn Interv Imaging 98:179–190 10.1016/j.diii.2017.01.001 28131457

[11] Portnow LH, Georgian-Smith D, Haider I et al (2022) Persistent inter-observer variability of breast density assessment using BI-RADS® 5th edition guidelines. Clin imaging 83:21–27 10.1016/j.clinimag.2021.11.034 PMC8857050 34952487

[12] Kshirsagar A (2020) Quantra™ 2.2 software design intent and clinical performance. Clinical Solutions, Research and Development, Hologic, Inc., Marlborough

[13] Youk JH, Gweon HM, Son EJ, Kim J-A (2016) Automated volumetric breast density measurements in the era of the BI-RADS fifth edition: a comparison with visual assessment. AJR Am J Roentgenol 206:1056–1062 10.2214/AJR.15.15472 26934689

[14] Holland R, Rijken H, Hendriks J (2007) The Dutch population-based mammography screening: 30-year experience. Breast Care 2:12–18

[15] van der Willik KD, Ruiter R, van Rooij FJ et al (2020) Ascertainment of cancer in longitudinal research: the concordance between the Rotterdam Study and the Netherlands Cancer Registry. Int J Cancer 147:633–640 10.1002/ijc.32750 PMC7317466 31642518

[16] van Engeland S, Snoeren PR, Huisman H, Boetes C, Karssemeijer N (2006) Volumetric breast density estimation from full-field digital mammograms. IEEE Trans Med Imaging 25:273–282 10.1109/TMI.2005.862741 16524084

[17] Fleiss JL, Levin B, Paik MC (2013) Statistical methods for rates and proportions. John Wiley & Sons

[18] Altman DG (1999) Some common problems in medical research. Practical Statistics For Medical Research. Chapman and Hall/CRC, Boca Raton

[19] Bangdiwala SI, Shankar V (2013) The agreement chart. BMC Med Res Methodol 13:1–7 10.1186/1471-2288-13-97 PMC3733724 23890315

[20] Kusumaningtyas N, Supit N, Murtala B et al (2024) A systematic review and meta-analysis of correlation of automated breast density measurement. Radiography 30:1455–1467 10.1016/j.radi.2024.08.003 39164186

[21] Brandt KR, Scott CG, Ma L et al (2016) Comparison of clinical and automated breast density measurements: implications for risk prediction and supplemental screening. Radiology 279:710–719 10.1148/radiol.2015151261 PMC4886704 26694052

[22] Youk JH, Gweon HM, Son EJ, Eun NL, Kim J-A (2021) Fully automated measurements of volumetric breast density adapted for BIRADS 5th edition: a comparison with visual assessment. Acta Radiol 62:1148–1154 10.1177/0284185120956309 32910685

[23] Morrish OW, Tucker L, Black R, Willsher P, Duffy SW, Gilbert FJ (2015) Mammographic breast density: comparison of methods for quantitative evaluation. Radiology 275:356–365 10.1148/radiol.14141508 25559234

[24] Schmachtenberg C, Hammann-Kloss S, Bick U, Engelken F (2015) Intraindividual comparison of two methods of volumetric breast composition assessment. Acad Radiol 22:447–452 10.1016/j.acra.2014.12.003 25586710

[25] van der Waal D, den Heeten GJ, Pijnappel RM et al (2015) Comparing visually assessed BI-RADS breast density and automated volumetric breast density software: a cross-sectional study in a breast cancer screening setting. PLoS One 10:e0136667 10.1371/journal.pone.0136667 PMC4559403 26335569

[26] Habel LA, Alexeeff SE, Achacoso N et al (2023) Examination of fully automated mammographic density measures using LIBRA and breast cancer risk in a cohort of 21,000 non-Hispanic white women. Breast Cancer Res 25:92 10.1186/s13058-023-01685-6 PMC10405373 37544983

[27] Eng A, Gallant Z, Shepherd J et al (2014) Digital mammographic density and breast cancer risk: a case-control study of six alternative density assessment methods. Breast Cancer Res 16:1–12 10.1186/s13058-014-0439-1 PMC4303120 25239205

[28] Astley SM, Harkness EF, Sergeant JC et al (2018) A comparison of five methods of measuring mammographic density: a case-control study. Breast Cancer Res 20:10 10.1186/s13058-018-0932-z PMC5799922 29402289

[29] Lee SE, Son N-H, Kim MH, Kim E-K (2022) Mammographic density assessment by artificial intelligence-based computer-assisted diagnosis: a comparison with automated volumetric assessment. J Digit Imaging 35:173–179 10.1007/s10278-021-00555-x PMC8921363 35015180

[30] Damases CN, Brennan PC, McEntee MF (2015) Mammographic density measurements are not affected by mammography system. J Med Imaging 2:015501 10.1117/1.JMI.2.1.015501 PMC4478994 26158085

[31] Gubern-Mérida A, Kallenberg M, Platel B, Mann RM, Martí R, Karssemeijer N (2014) Volumetric breast density estimation from full-field digital mammograms: a validation study. PLoS One 9:e85952 10.1371/journal.pone.0085952 PMC3897574 24465808

[32] Nederend J, Duijm LE, Louwman MW et al (2014) Impact of the transition from screen-film to digital screening mammography on interval cancer characteristics and treatment—a population based study from the Netherlands. Eur J Cancer 50:31–39 10.1016/j.ejca.2013.09.018 24275518

