# Supplementary material for: Comparison of three algorithms to measure breast density on mammograms in a population-based screening cohort
Source: Eur Radiol. 2026 Jun 10;36(11):8347–58. doi: 10.1007/s00330-026-12681-7 (PMC13661706; doi:10.1007/s00330-026-12681-7)
Supplement: Supplementary file 1 [file 330_2026_12681_MOESM1_ESM.pdf]

Comparison of three algorithms to measure breast density on mammograms in a population-based screening cohort

ELECTRONIC SUPPLEMENTARY MATERIAL

Supplementary results

Table S1: Associations of breast density measures with invasive breast cancer risk and five-year area under the receiver operating curves to discriminate between participants with and without invasive breast cancer. *Continuous measures were log-transformed and normalized.*

		Associations with breast cancer risk		*Breast cancer vs. no breast cancer*	
**Breast density measure**		**Hazard ratio**	**95%CI**	**Area under the curve**	**95%CI**
**VDG4**	**A**	0.78	0.64-0.96	0.53	0.51-0.55
	**B**	ref	ref		
	**C**	1.19	0.98-1.44		
	**D**	1.43	1.08-1.87		
**VDG5**	**A**	0.71	0.52-0.96	0.53	0.51-0.55
	**B**	ref	ref		
	**C**	1.23	1.4-1.47		
	**D**	1.42	1.11-1.83		
**Quantra**	**A**	0.75	0.57-0.99	0.55	0.52-0.57
	**B**	ref	ref		
	**C**	1.39	118-1.65		
	**D**	1.56	1.17-2.06		
**iCAD**	**A**	0.82	0.67-1.02	0.53	0.51-0.55
	**B**	ref	ref		
	**C**	1.37	1.15-1.4		
	**D**	1.53	1.05-2.22		
**VBD % (Volpara)**	**%**	1.21	1.12-1.30	0.56	0.54-0.58
**Max. VBD % (Volpara)**	**%**	1.22	1.14-1.31	0.56	0.54-0.59
**Percent dense tissue % (iCAD)**	**%**	1.27	1.18-1.38	0.57	0.55-0.59
